# Small Molecule Modulators of RING-Type E3 Ligases: MDM and Cullin Families as Targets

**DOI:** 10.3389/fphar.2018.00450

**Published:** 2018-05-08

**Authors:** Emil Bulatov, Almaz Zagidullin, Aygul Valiullina, Regina Sayarova, Albert Rizvanov

**Affiliations:** ^1^Institute of Fundamental Medicine and Biology, Kazan Federal University, Kazan, Russia; ^2^A.E. Arbuzov Institute of Organic and Physical Chemistry, Kazan Scientific Center, Russian Academy of Sciences, Kazan, Russia

**Keywords:** ubiquitin–proteasome system, RING-type E3 ligases, MDM family, Cullin family, small molecules, induced protein degradation, PROTACs, SNIPERs

## Abstract

Ubiquitin–proteasome system (UPS) is a primary signaling pathway for regulation of intracellular protein levels. E3 ubiquitin ligases, substrate-specific members of the UPS, represent highly attractive protein targets for drug discovery. The importance of E3 ligases as prospective targets for small molecule modulation is reinforced by ever growing evidence of their role in cancer and other diseases. To date the number of potent compounds targeting E3 ligases remains rather low and their rational design constitutes a challenging task. To successfully address this problem one must take into consideration the multi-subunit nature of many E3 ligases that implies multiple druggable pockets and protein–protein interfaces. In this review, we briefly cover the current state of drug discovery in the field of RING-type E3 ligases with focus on MDM and Cullin families as targets. We also provide an overview of small molecule chimeras that induce RING-type E3-mediated proteasomal degradation of substrate proteins of interest.

## Introduction

In the last two decades, scientists around the globe paid increasing attention toward UPS, a primary pathway for regulation of protein turnover and removal of misfolded proteins in eukaryotic cells. The molecular mechanism of proteasomal protein degradation is driven by a consecutive action of three enzyme categories (E1 activating, E2 conjugating, and E3 ligating) that covalently tag substrate proteins with a chain of ubiquitins, small regulatory proteins (Hershko and Ciechanover, 1998; Pickart, 2001). Subsequent fate of the substrate is defined depending on type of ubiquitin linkage, its length and post-translational modifications. UPS operates in nucleus and cytoplasm, which enables ubiquitin-mediated regulation of cell cycle control, DNA damage response, innate immunity, and proteasomal protein degradation.

E3 ligases confer substrate specificity of the whole UPS and function via two primary mechanisms – they can play an intermediary catalytic role in transfer of ubiquitins from E2∼Ub conjugate to the substrate or, alternatively, facilitate direct ubiquitin transfer bypassing the E3 ligase itself. The first mechanism is common for Homologous to E6-AP Carboxy Terminus (HECT)-type E3s, whereas the second one is typical for RING-type E3s.

Initially the RING domain was identified in RING1 protein and later confirmed to be a structural element of Rbx1 that functions as E2-recruiting subunit of E3 ligase (Kamura et al., 1999; Ohta et al., 1999; Seol et al., 1999; Tan et al., 1999). The canonical RING finger motif can be represented as Cys-X_2_-Cys-X_(9-39)_-Cys-X_(1-3)_-His-X_(2-3)_-Cys-X_2_-Cys-X_(4-48)_-Cys-X_2_-Cys, where X is any other amino acid (Deshaies and Joazeiro, 2009). The domain includes two Zn^2+^-coordinated loops and intervening central α-helix that together form a conserved structural platform for anchoring the E2 conjugating enzyme.

RING E3s can be categorized according to the form of subunit organization: (1) mono-subunit ligases like MDM and Cbl; (2) multi-subunit complexes such as CRL, anaphase promoting complex/cyclosome (APC/C) (Castro et al., 2005) and FANC (Metzger et al., 2014). Another important RING family members are RBRs, the single subunit enzymes with multiple RING domains (Spratt et al., 2014). In addition, worth mentioning are U-box ligases, containing atypical RING domains without coordinated Zn^2+^ ions, that are often featured as separate from canonical RING E3s (Hatakeyama et al., 2001).

**FIGURE 1:**
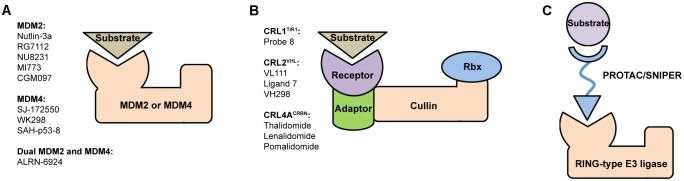
Structural organization of **(A)** MDM and **(B)** CRL RING-type E3 ubiquitin ligases and a brief list of their most potent modulators. MDM2 and MDM4 are single subunit proteins containing C-terminal RING finger domain. In contrast, CRLs are constituted of several subunits, such as receptor, adaptor, Cullin scaffold and RING-box protein. **(C)** Schematic representation of PROTAC/SNIPER molecule acting to induce ubiquitination and subsequent proteasomal degradation of substrate protein of interest. These small molecule degraders contain substrate-specific component, short linker, and E3-specific component.

Notably, E3 ligases were proposed to become the “new kinases” owing to the significant therapeutic and market potential of their small molecule inhibitors (Cohen and Tcherpakov, 2010). Recent advances demonstrated that E3s in general, and MDM and Cullin families (**Figures 1A,B**), in particular, have captured significant attention of the research community due to their key role in poly-ubiquitination of substrate proteins (Maniaci et al., 2017).

Here, we briefly highlight members of MDM and CRL families of RING-type E3 ligases, their primary functions and small molecule modulation.

## MDM Family – MDM2 and MDM4

MDM family includes MDM2 and its homolog MDM4 (also known as MDMX). Both enzymes are overexpressed in many cancers and function as key negative regulators of oncosuppressor protein p53 that is often referred to as “guardian of the genome” (Haupt et al., 2017). MDM2-MDM4-p53 signaling circuit regulates core metabolic pathways that involve DNA damage response, induction of apoptosis, cell cycle arrest, and senescence. In nearly half of cancers p53 is downregulated due to overexpression of MDM2 or/and MDM4, while the other half is caused by tumorigenic missense mutations of p53, mostly occurring in DNA-binding domain (Muller and Vousden, 2013).

MDM2 binds wild-type p53 with high affinity, blocks its transcriptional activator functions and regulates its nuclear export, stability, intracellular levels (Geyer et al., 2000). MDM4 shares a high structural homology with MDM2, mainly in the N-terminal p53-binding domain, yet due to low E3 ligase activity its overexpression does not lead to dramatic p53 downregulation (Huang et al., 2011). Interaction of MDM2 and MDM4 via their RING domains results in formation of MDM2/MDM4 hetero-dimeric complex that enhances p53 ubiquitination and degradation (Pant et al., 2011).

Many therapeutic strategies based on disrupting p53/MDM2 protein–protein interaction are aimed to stabilize and activate p53 in order to initiate programmed cell death in cancers with overexpressed MDM2 (Zhang et al., 2015). Numerous examples of MDM2 inhibitors include small synthetic molecules such as Nutlin-3a (Vassilev et al., 2004), RG7112 (Vu et al., 2013), NU8231 (Hardcastle et al., 2011), MI773 (Zhao et al., 2013), CGM097 (Holzer et al., 2015) with several others already in clinical trials as drug candidates (Tisato et al., 2017) (**Figure 1A**). Development of potent MDM2 inhibitors remains a longstanding mainstream in the p53 field, meanwhile the importance of therapeutic targeting MDM4 was recognized later. To date a certain progress was achieved in development of p53/MDM4 inhibitors such as SJ-172550 (Reed et al., 2010; Bista et al., 2012), WK298 (Popowicz et al., 2010) and stabilized peptide SAH-p53-c8 (Bernal et al., 2010).

In addition, simultaneous inhibition of both MDM2 and MDM4 enzymes, i.e., by means of dual inhibitors, was proposed to enhance p53 activation (Hu et al., 2007; Popowicz et al., 2010; Zaytsev et al., 2015). Stapled peptide ALRN-6924 (Aileron, Inc.) is an example of dual inhibitor that is currently undergoing clinical trials against solid tumors and lymphomas (Chang et al., 2013). Also, worth mentioning an approach based on targeting MDM2/MDM4 interface with short peptides that initiate p53-induced apoptosis and slowdown of tumor growth (Pellegrino et al., 2015).

## Cullin Family – Cullin Ring E3 Ligases (CRLs)

Cullin RING E3 ligases constitute the largest family of E3 ligases with over 200 known members (Sarikas et al., 2011). In certain cell types up to 20% of the proteasomal protein degradation is mediated by CRLs (Soucy et al., 2009). Classification of CRL family is based on type of Cullin scaffold serving as a backbone of the multi-subunit protein complex. The evolutionary conserved Cullin protein has several forms that share structural similarity – Cul1, Cul2, Cul3, Cul4A, Cul4B, Cul5, and Cul7. Accordingly, the resulting E3 ligases are named CRL1-CRL7. Some classifications also include Cul9 known as p53-associated parkin-like cytoplasmic protein (PARC) (Li and Xiong, 2017).

Assembly of the multi-subunit CRLs was originally reported for the archetypal Skp1-Cul1-Rbx1 complex (Feldman et al., 1997). The CRL structure is based on modular organization of constituent subunits such as substrate receptors (F-box, SOCS-box, DCAF, and BTB), adaptors (Skp1, ElonginB, ElonginC, DDB1, and BTB), Cullin scaffolds (Cul1-Cul7 and Cul9) and RING finger proteins (Rbx1 and Rbx2). The wide range of building blocks and their combinations enables formation of a multitude of functionally diverse E3 ligases (Bosu and Kipreos, 2008; Sarikas et al., 2011; Lydeard et al., 2013). Full-size CRLs are notoriously difficult to obtain and characterize, to date only several complexes containing a complete set of subunits were reported, including SOCS2-EloB-EloC-Cul5-Rbx2 (Bulatov et al., 2015), VHL-EloB-EloC-Cul5-Rbx1 (Cardote et al., 2017), Skp2-Skp1-Cul1-Rbx1 (Zheng et al., 2002), DDB2-DDB1-Cul4A-Rbx1 (Fischer et al., 2011).

Drug discovery in the CRL field keeps advancing, driven by foreseeable role of these enzymes as targets in numerous human diseases (Petroski, 2008; Cohen and Tcherpakov, 2010; Andérica-Romero et al., 2013; Zhao and Sun, 2013). The CRLs as drug targets are promising, yet highly sophisticated due to diverse putative combinations of structural components that result in multiple protein–protein interfaces.

The growing number of reported CRL crystal structures includes full-size enzymes, individual components and their complexes. Here, particularly important are structures of receptor subunits with bound small molecules because they provide crucial information for rational design of potent modulators. Examples (**Figure 1B**) include Probe 8, auxin-mimic that disrupts interaction between TIR1 (receptor in CRL1^TIR1^) and its substrate Aux/IAA (Hayashi et al., 2008); VL111, Ligand 7 and their enhanced analog VH298 that inhibit binding of VHL protein (receptor in CRL2^VHL^) to substrate hypoxia-inducible factor 1-alpha (HIF1α) (Buckley et al., 2012; Galdeano et al., 2014; Soares et al., 2018); thalidomide and its derivatives lenalidomide, pomalidomide that modulate CRL4A^CRBN^ activity by binding to its CRBN receptor subunit (Chamberlain et al., 2014; Fischer et al., 2014).

Targeting CRLs for therapeutic applications is obstructed by several factors. Firstly, there is no general approach and each enzyme has to be tackled individually according to its structural and functional characteristics. Secondly, CRLs are complex molecular entities assembled from several independent subunits that form a number of protein–protein interfaces and druggable pockets. Surprisingly, the apparent complexity of this multi-subunit system might turn out to be an advantage – the multitude of interfaces and pockets provides additional opportunities for design of specific binders (Bulatov and Ciulli, 2015). Small molecules modulate CRL activity directly by inhibiting substrate/receptor interaction, disrupting CRL assembly, inducing allosteric conformational shifts that lead to suppressed activity or altered ensemble dynamics (Lee and Craik, 2009). In some cases modulators stabilize specific protein–protein interactions within the CRL complex (Thiel et al., 2012).

## Ring-Type E3 Ligases as Targets for Drug Discovery

Drug discovery in the UPS field thrived since FDA approval of 26S proteasome inhibitors Bortezomib (approved in 2003, marketed as Velcade^®^) and more recently Carfilzomib (approved in 2012, marketed as Kyprolis^®^) for treatment of multiple myeloma. Although proteasome inhibitors demonstrate selectivity toward tumor cells and lead to apoptosis, they also suppress proteasome-mediated degradation of all intracellular proteins resulting in potential side effects (Shibata et al., 2018). In addition to that, many patients were demonstrated to develop resistance toward Bortezomib that acts as a reversible competitive inhibitor of 26S proteasome (Ohoka et al., 2018). Strategies to overcome the resistance include design of inhibitors with alternative mechanism of action, i.e., Carfilzomib covalently binds proteasome and causes its irreversible inhibition.

Another emerging and highly promising strategy is to target different levels of ubiquitination cascade upstream of proteasome, mainly E1, E2, and E3 enzymes (Shibata et al., 2017). Here, E3 ligases determine substrate specificity and, therefore, represent primary molecular targets for small molecule modulation. Development of potent E3 ligase modulators is risky and challenging task complicated by diverse protein–protein interfaces of the multi-component complexes, lack of a classical enzymatic/catalytic active site and specificity problems stipulated by a variety of potential substrates.

RING-type E3 ligases were previously successfully targeted using small molecule inhibitors of protein–protein interactions, i.e., binding at substrate/receptor HIF1α/VHL (Ohoka et al., 2017b), p53/MDM2 and p53/MDM4 interfaces (Ohoka et al., 2017a), adaptor/receptor interface Skp1/Skp2 (Chen et al., 2008) and several other, as reviewed in Bulatov and Ciulli (2015).

In addition, it would be reasonable to mention an auxiliary protein NEDD8 that serves as crucial regulator of CRL functions (Soucy et al., 2009). The ubiquitin-like protein NEDD8 gets covalently conjugated to a specific conserved lysine residue at C-terminal domain of Cullin scaffold in a process termed NEDDylation. NEDD8 conjugation enhances substrate ubiquitination by promoting CRL dynamics and inducing conformational shift of the protein ensemble that brings together E3-bound substrate and Rbx–E2∼Ub (Duda et al., 2008, 2011). In addition, MDM2 was demonstrated not only to autoNEDDylate itself, but also to mediate p53 NEDDylation that inhibits its transcriptional activity (Xirodimas et al., 2004). Pevonedistat (also known as MLN4924), a first-in-class selective inhibitor of NEDD8-activating enzyme (NAE) that mediates NEDDylation, is currently undergoing several clinical trials against different types of cancer (Swords et al., 2015).

## Targeted Protein Degradation Mediated by Ring-Type E3 Ligases

A novel paradigm-shifting approach that dramatically increases the attractiveness of E3 ligases as targets for drug design is based on proteolysis-targeting chimeras (PROTACs) (Buckley and Crews, 2014; Lai and Crews, 2017). These are hetero-bifunctional compounds with bivalent selectivity, they consist of three key elements: substrate-specific component (“warhead”), short linker, and E3-specific component. PROTACs bind and bring into close proximity substrate protein of interest (POI) and E3 ligase thus facilitating E3-mediated ubiquitination of the substrate (**Figure 1C**). This approach, sometimes called as “chemical knockdown,” enables ligand-induced degradation of specific endogenous proteins (Bondeson et al., 2015).

Historically the first small-molecule-based PROTAC degrader was developed for recruiting MDM2 E3 ligase by Nutlin-3a imidazoline derivative, as reported by Crews laboratory (Schneekloth et al., 2008). In this study androgen receptor (AR) was targeted by SARM compound connected to Nutlin-3a moiety via PEG-based linker. Treatment of AR-expressing HeLa cells with SARM-Nutlin-3a bivalent molecule resulted in depletion of AR levels. Since then there were no subsequent reports of MDM2-mediated PROTACs, though given the large and still expanding repertoire of potent MDM2 inhibitors it would be reasonable to expect much higher interest in such degraders.

CRL2^VHL^ is the most prominent example of E3 ligase directed by PROTACs to ubiquitinate a recruited substrate protein and result in its proteasomal degradation. The E3-specific component is designed to mimic HIF1α, natural substrate of VHL receptor, post-translationally hydroxylated at prolines 402 and/or 564. Examples of proteins selectively targeted by PROTACs via CRL2^VHL^-mediated mechanism include BET family of epigenetic regulators BRD2, BRD3, and BRD4 (Zengerle et al., 2015; Raina et al., 2016; Gadd et al., 2017; Zhou et al., 2017; Chan et al., 2018), steroid hormone receptor ERRα and serine-threonine kinase RIPK2 (Bondeson et al., 2015). Another recent study demonstrated successful degradation of oncogenic tyrosine kinase BCR-ABL using both CRL2^VHL^- and CRL4A^CRBN^-specific PROTACs (Lai et al., 2016). Here, FDA-approved tyrosine kinase inhibitors Bosutinib and Dasatinib served as PROTAC “warheads,” whereas hydroxy-proline derivative and pomalidomide were used as CRL2^VHL^- and CRL4A^CRBN^-binding components, respectively. Similarly, PROTAC-directed CRL4A^CRBN^-mediated target degradation also includes BRD4 and cytosolic protein FKBP12 (Lu et al., 2015; Winter et al., 2015). In addition, Ciulli laboratory recently reported curious example of the first homo-bivalent PROTAC (Homo-PROTAC) that simultaneously recruits two CRL2^VHL^ molecules and induces their suicidal self-degradation (Maniaci et al., 2017). Highly potent Homo-PROTAC CM11 contains two molecules of the previously described VH298 compound connected via polyethylene glycol linker (Frost et al., 2016; Soares et al., 2018).

SNIPERs, an abbreviated form of specific and non-genetic IAP-dependent protein erasers, are a class of small-molecule degraders similar to PROTACs (Itoh et al., 2010, 2011). These compounds recruit IAP family of RING-type E3 ligases – cIAP1, cIAP2, and XIAP. The SNIPER chemical structure consists of selective IAP antagonist (i.e., Bestatin, MV1, and LCL161), PEG linker and peptide- or small-molecule-based POI-specific component. The initial concept was further explored by Naito laboratory that recently demonstrated selective proteasomal degradation of a range of substrate proteins, including ERα (Ohoka et al., 2018), BCR-ABL (Shibata et al., 2017), BRD4 and PDE4 (Ohoka et al., 2017b), NOTCH1 (Ohoka et al., 2017a), CRABP-II (Okuhira et al., 2011).

These examples, listed in **Table 1**, illustrate that induced protein depletion mediated by small molecule degraders (PROTACs and SNIPERs) specific for certain RING-type E3 ligases (MDMs, CRLs, and IAPs) is a justified paradigm for drug discovery. These compounds operate in sub-stoichiometric manner and catalyze enzymatic removal of substrate POIs (chemical knockdown) rather than their inhibition via binding site occupation. The PROTAC/SNIPER-based approach counteracts restoration of intracellular POI levels and is seen as a promising addition to conventional gene knockdown techniques (i.e., RNAi, antisense oligonucleotides, and CRISPR/Cas9) (Crews, 2018).

**Table 1:** List of substrate proteins targeted by PROTACs and SNIPERs for RING-type E3-mediated proteasomal degradation.

Substrate	RING-type E3 ligase	Reference
**PROTACs**		
AR	MDM2	Schneekloth et al., 2008
BRD2, BRD3, BRD4	CRL2^VHL^	Thiel et al., 2012; Zengerle et al., 2015; Raina et al., 2016; Gadd et al., 2017; Chan et al., 2018
ERRα, RIPK2	CRL2^VHL^	Bondeson et al., 2015
VHL	CRL2^VHL^	Maniaci et al., 2017
BCR-ABL	CRL2^VHL^, CRL4A^CRBN^	Lai et al., 2016
FKBP12	CRL4A^CRBN^	Winter et al., 2015
BRD4	CRL4A^CRBN^	Lu et al., 2015; Winter et al., 2015
**SNIPERs**		
AR	cIAP1/cIAP2/XIAP	Shibata et al., 2018
ERα		Ohoka et al., 2018
BCR-ABL		Shibata et al., 2017
BRD4		Ohoka et al., 2017b
PDE4		Ohoka et al., 2017b
NOTCH1		Ohoka et al., 2017a
CRABP-II		Okuhira et al., 2011


## Conclusion

Many E3 ubiquitin ligases are implicated in cellular physiology and homeostasis at multiple regulatory levels, play crucial roles in a wide range of human diseases, including cancer and inflammatory disorders. The RING-type ligases represent the largest class of E3s in humans and are responsible for recognition, poly-ubiquitination and subsequent proteasomal degradation of numerous substrate proteins. Given the structural intricacy of RING-type E3s and their diverse functions it is essential to understand molecular principles that govern assembly and interactions of subunits within the protein complex. We anticipate a forthcoming re-focusing of research efforts from structural/functional characterization of these enzymes to rational design of precision molecular tools capable of modulating E3 activity in living cells and organisms. One could envisage that targeted therapeutic action upstream of proteasome could potentially lead to emerging resistance caused by feedback-driven compensatory mechanisms. This could potentially be overcome by administering a synergistic combination of drugs that act via distinct molecular mechanisms.

The MDM and CRL families of RING-type E3s are growing in importance as attractive targets for small molecule therapeutics that could operate not only as inhibitors but also activators of enzymatic activity, disruptors and stabilizers of protein–protein interactions, regulators of protein dynamics. The availability of X-ray structural data, especially for full-size enzymes, remains to be one the main limitations for structure-based design of modulators for multi-subunit E3 complexes. Nevertheless, slow yet steady increase of solved crystal structures of RING-type E3s and their ligand-bound complexes provides opportunities for further rational design of highly potent modulators. General strategies for discovery of novel E3 modulators include *in vitro* screening of compound libraries using functional assays (i.e., ubiquitination assay) (Sun, 2005); software packages (i.e., ICM-Pocket Finder) predicting druggable pockets for subsequent *in silico* ligand screening and docking (Cardozo and Abagyan, 2005); fragment-based discovery and rational structure elaboration (Van Molle et al., 2012; Gadd et al., 2015).

In addition, PROTAC/SNIPER-induced ubiquitination and intracellular degradation of specific substrates offers an added layer of specificity and constitutes an emerging strategy for post-translational regulation of protein levels. These compounds demonstrate high potential for a novel modality of chemical intervention on UPS and hold much promise as prospective therapeutic candidates. Such chemical protein knockdown technologies based on RING-type E3 modulation still remain largely untapped by the pharmaceutical industry, however, it is reasonable to expect a major progress in this direction in near future.

## Author Contributions

EB wrote introduction, conclusion, sections about Cullin family and targeted protein degradation. AV and RS wrote section about MDM family. AZ and AR wrote section about drug discovery.

## Conflict of Interest Statement

The authors declare that the research was conducted in the absence of any commercial or financial relationships that could be construed as a potential conflict of interest.

## Abbreviations

ABL: Abelson tyrosine-protein kinase
AR: androgen receptor
BCR: breakpoint cluster region protein
BET: bromodomain and extra-terminal domain
BRD2: bromodomain-containing protein 2
cIAP1: cellular inhibitor of apoptosis protein 1
CRABP-II: cellular retinoic acid binding protein-II
CRL: cullin RING E3 ligase
ERα: estrogen receptor α
FANC: Fanconi anemia ligase
HIF1α: hypoxia-induced factor 1-α
IAP: inhibitor of apoptosis protein
MDM2: murine double minute 2
NAE: NEDD8-activating enzyme
NEDD8: neural precursor cell expressed developmentally down-regulated protein 8
NOTCH1: neurogenic locus notch homolog protein 1
PDE4: cAMP-specific 3′,5′-cyclic phosphodiesterase 4
POI: protein of interest
PROTAC: proteolysis-targeting chimera
RBR: RING-between RING-RING
RING: really interesting new gene
SARM: selective androgen receptor modulator
SNIPER: specific and non-genetic IAP-dependent protein erasers
UPS: ubiquitin–proteasome system
VHL: von Hippel-Lindau
XIAP: X-linked inhibitor of apoptosis protein
